# Machine Learning Predicts Non-Preferred and Preferred Vertebrate Hosts of Tsetse Flies (*Glossina spp*.) Based on Skin Volatile Emission Profiles

**DOI:** 10.1007/s10886-025-01582-6

**Published:** 2025-03-07

**Authors:** Olabimpe Y. Orubuloye, David P. Tchouassi, Abdullahi A. Yusuf, Christian W. W. Pirk, Daniel K. Masiga, Edward Kariuki, Baldwyn Torto

**Affiliations:** 1 International Centre of Insect Physiology and Ecology (icipe), P.O. Box 30772-00100, Nairobi, Kenya; 2 Department of Zoology and Entomology, University of Pretoria, Private Bag X20, Hatfield, Pretoria, 0028 South Africa; 3 Veterinary and Capture Service Department, Kenya Wildlife Service (KWS), Nairobi, Kenya

**Keywords:** African trypanosomosis, Olfaction, Genus *Glossina*, Skin odors, Wildlife, Gas chromatography/mass spectrometry

## Abstract

Tsetse fly vectors of African trypanosomosis preferentially feed on certain vertebrates largely determined by olfactory cues they emit. Previously, we established that three skin-derived ketones including 6-methyl-5-hepten-2-one, acetophenone and geranyl acetone accounted for avoidance of zebra by tsetse flies. Here, we tested the hypothesis that these three ketones serve as biomarkers for tsetse flies to distinguish between non-preferred- and preferred-vertebrate hosts. We used coupled gas chromatography/mass spectrometry to analyze and compare the skin volatile emissions of two non-preferred- (waterbuck and zebra) and four preferred- (buffalo, donkey, horse, warthog) vertebrate hosts in two wildlife parks in Kenya. We detected a total of 96 volatile organic compounds (VOCs) in the skin emissions composed mainly of aldehydes, ketones, alcohols, phenols and alkanes, which varied with the vertebrate host. Using random forest analysis, we found a weak correlation between the three skin-odor repellent ketones and non-preferred and preferred vertebrate hosts. However, we found that the three repellent ketones plus skin background odors may be more sensitive chemical signals for tsetse flies to discriminate vertebrate hosts. These results contribute to understanding tsetse fly vertebrate host preferences in their natural habitat across geographic scales.

## Introduction

The Afro-endemic tsetse flies *Glossina spp*. (Diptera: Glossinidae) are obligate blood feeding insects and vectors of trypanosome pathogens causing African trypanosomosis, a devastating but neglected tropical disease. African trypanosomosis affects both humans and animals, particularly livestock, with 60 million and 50 million humans and cattle, respectively, at risk of infection in 37 sub-Saharan African countries (FAO 2024). While human African trypanosomosis is a declining public health concern (FAO and WHO 2022; Franco et al. 2024), animal African trypanosomosis still poses a constraint to sustainable agricultural and livestock production accounting for about three million cattle deaths and losses valued at USD 4.75 billion annually (Abro et al. 2023; FAO 2024; Muriithi et al. 2021; Shaw et al. 2017). There are 31 extant species and sub-species of tsetse flies within the *Glossina* species complex classified into three groups, namely: savannah or *morsitans* (subgenus *Glossina* s.s.), riverine or *palpalis* (subgenus *Nemorhina*) and forest or *fusca* (subgenus *Austenina*) group (Orubuloye et al. 2024; Vreysen et al. 2013), which differ in their habitat requirements, host preferences, epidemiological and economic importance.

Like other blood feeding insects, such as mosquitoes (Takken 1991) and sandflies (Tchouassi et al. 2024), tsetse flies also locate their hosts using a combination of olfactory and visual cues. Visual cues are important at close range for landing decisions (Gibson and Torr 1999; Vreysen et al. 2013), but tsetse flies principally employ olfactory cues to locate and discriminate vertebrate hosts for a blood meal (Gikonyo et al. 2000, 2003; Olaide et al. 2019; Takken and Knols 2010). Host-derived olfactory cues comprise kairomones (attractants) and allomones (repellents), mainly volatile organic compounds (VOCs) suited for long range communication. For example, the VOCs 3-n-propylphenol and 4-methylphenol (p-cresol) emanating from host urine, and 1-octen-3-ol and acetone from host breath are important kairomones for tsetse host location (Masiga et al. 2014; Rayaisse et al. 2010; Vale and Torr 2004). A four-component blend of these attractive chemicals combined with visual traps or targets form the basis of the bait technology, an effective and highly successful semiochemical-based control tool for savannah tsetse flies (Masiga et al. 2014).

Selective feeding on certain vertebrates by tsetse flies is well described in the literature (Auty et al. 2016; Channumsin et al. 2021; Clausen et al. 1998; Ebhodaghe et al. 2021; Gashururu et al. 2023; Makhulu et al. 2021; Moloo et al. 1993; Muturi et al. 2011) and driven mainly by semiochemicals which are now being exploited for their control (Bett et al. 2015; Gikonyo et al. 2003; Orubuloye et al. 2024; Saini et al. 2017). Generally, vertebrates like buffalo, cattle, warthog, elephant, giraffe are more frequently fed on by savannah tsetse flies compared to others such as waterbuck, zebra, wildebeest, impala and Thompson’s gazelle. For instance, in the Masai Mara National Reserve Kenya (Auty et al. 2016) and Serengeti National Park Tanzania (Makhulu et al. 2021) both in eastern Africa, the savannah tsetse flies *G. pallidipes* and *G. swynnertoni* preferentially fed on vertebrates such as African buffalo, warthog and elephant compared to the more abundant wildebeest and zebra. Similar feeding preferences have been reported for tsetse flies in areas of Kenya, Tanzania and Uganda in East Africa (Channumsin et al. 2021; Ebhodaghe et al. 2021; Muturi et al. 2011), Zambia in southern Africa (Gaithuma et al. 2020), and Rwanda, Central Africa (Gashururu et al. 2023). However, these feeding preferences could vary depending on the tsetse fly group. For instance, savannah tsetse flies are more selective than their riverine counterparts which are opportunistic feeders (Oloo et al. 2014; Torr and Vale 2015; Vale et al. 2014). Additionally, hunger status, environmental conditions (e.g. host availability), and species-specific preferences (Clausen et al. 1998; Gikonyo et al. 2000; Muturi et al. 2011; Orubuloye et al. 2024) may all contribute to the feeding preferences of tsetse flies.

Repellent chemicals emitted in the skin odors of non-preferred vertebrates but absent in the preferred ones, or found at sub-optimal amounts are important semiochemical drivers of tsetse fly feeding preferences (Gikonyo et al. 2002, 2003; Olaide et al. 2019). For example, a four-component tsetse repellent blend comprised of geranyl acetone, guaiacol, pentanoic acid and δ-octalactone and identified from the waterbuck protects livestock host (cattle) from tsetse bites and trypanosomosis infection (Bett et al. 2015; Saini et al. 2017). In another study, zebra skin odors, comprised of a blend of three ketones, 6-methyl-5-hepten-2-one, acetophenone, and geranyl acetone repelled two tsetse fly species *Glossina pallidipes* and *G. fuscipes fuscipes* (Olaide et al. 2019). Of the seven skin-derived repellents comprised of four classes of chemicals (ketone, phenol carboxylic acid and lactone), keto-compounds dominate (geranyl acetone, 6-methyl-5-hepten-2-one and acetophenone) with geranyl acetone being common to the skin-odors of waterbuck and zebra.

Hence, in this study, we tested the hypothesis that the zebra-derived ketones geranyl acetone, 6-methyl-5-hepten-2-one and acetophenone discriminate tsetse fly non-preferred and preferred hosts in two wildlife natural habitats in Kenya: Nguruman and Amboseli National Park. To achieve this, we screened skin volatile emission profiles and assessed the levels and ratios of the three ketones from selected tsetse fly non-preferred (waterbuck and zebra) and preferred (buffalo, donkey, horse, warthog) hosts. Data was further analyzed using machine learning algorithms to identify discriminatory skin odors among the vertebrates.

## Methods

### Study Area and Vertebrate Species

**Fig. 1:**
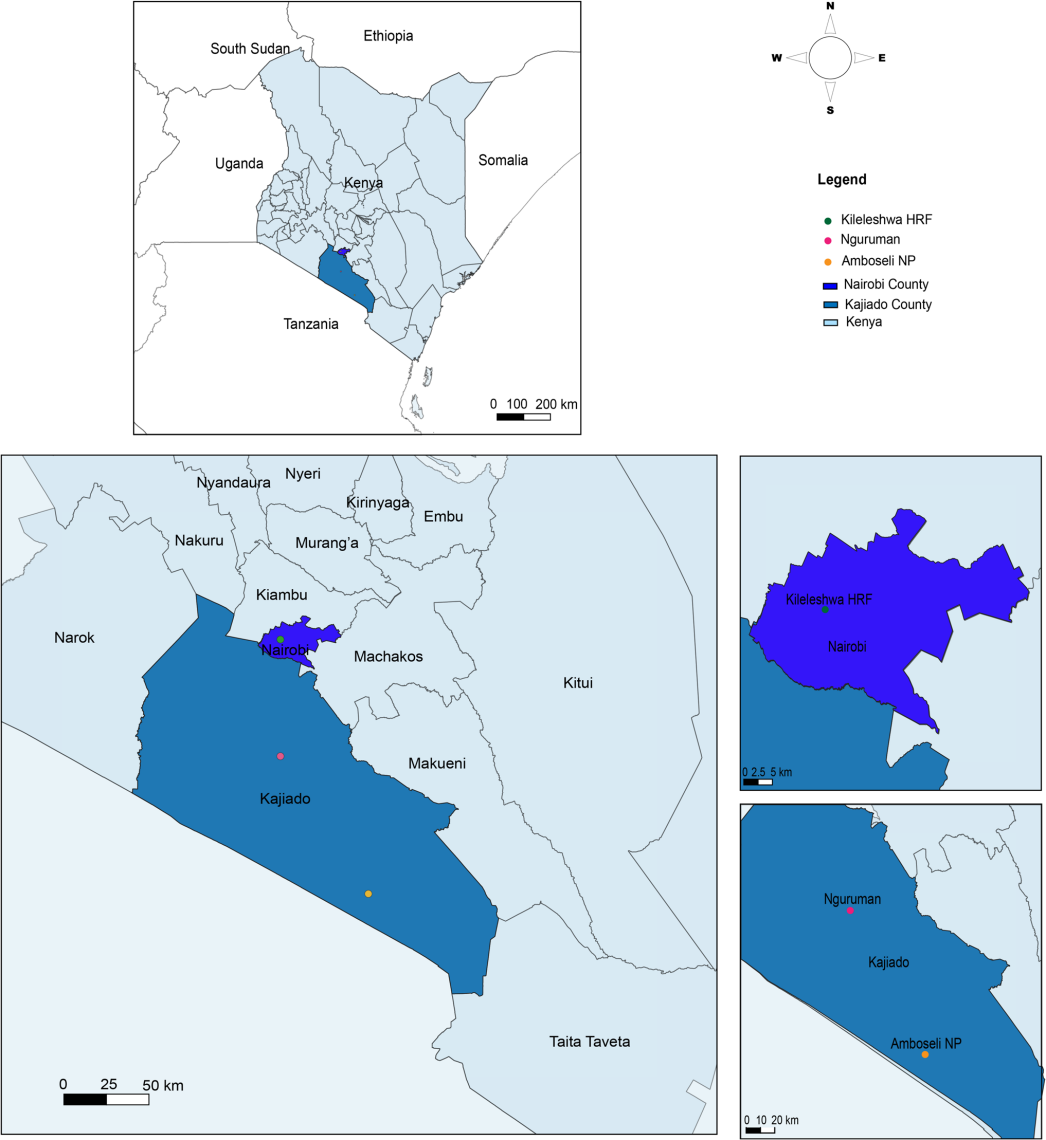
Map of Kenya showing the vertebrate skin odor collection areas. The maps were designed using the open-source software QGIS 3.20.3 “Odense” (https://www.qgis.org/download/). The coordinates of Nguruman, Amboseli National Park, and Kileleshwa horse riding farm (HRF) were obtained in the field using a GPS gadget (garmin etrex 20, https://buy.garmin.com/en-US/US/p/518046). The spatial data (Kenya, World) were downloaded from GADM (https://gadm.org/data.html), also an open source. Skin odors of zebra (*Equus quagga*) and donkey (*Equus africanus asinus*) were collected from Nguruman while waterbuck (*Kobus ellipsiprymnus defassa*), buffalo (*Syncerus caffer*), warthog (*Phacochoerus africanus*), and zebra (*Equus quagga*) were sampled from Amboseli, and horse (*Equus ferus caballus*) from Kileleshwa HRF in Nairobi, based on accessibility of the vertebrates in each location. For each vertebrate species, skin odor samples were collected from five adult individuals

This study was conducted between April 2017 and May 2019, in Nguruman and Amboseli National Park both of which are in Kajiado County, Kenya (Fig. 1). Amboseli National Park (S 2° 38′ 29″ E 37° 14′ 53″) is a 392 km^2^ protected area and highly diverse ecosystem famous for its large herds of elephants and other key wildlife species like buffalo, zebra, wildebeest, giraffe, a variety of antelopes and predators, and many bird species (KWS 2024; UNESCO 2024). Nguruman (S 1° 54′ 26.1″ E 36° 46′ 56.5) also supports diverse wildlife species which commonly interact with livestock (especially cattle, goat, and sheep) due to the proximity of Maasai pastoralists. Both areas harbor the savannah tsetse fly species *G. pallidipes*; although Nguruman provides more favourable breeding habitat for this species and the forest species *G. longipennis* (Olaide et al. 2019; Ouma et al. 2006). The study areas were selected based on accessibility of the target vertebrates, and the presence of savannah tsetse flies which are selective blood feeders.

Vertebrate species sampled included zebra (*Equus quagga*), waterbuck (*Kobus ellipsiprymnus defassa*), buffalo (*Syncerus caffer*), donkey (*Equus africanus asinus*), horse (*Equus ferus caballus*), and warthog (*Phacochoerus africanus*) were used in this study. Zebra and donkey were sampled in Nguruman, while waterbuck, buffalo, warthog and zebra were sampled in Amboseli, based on their accessibility in each location. Horse was not available in both localities and was sampled in Kileleshwa horse riding farm (S 1° 17′ 2.5″ E 36° 46′ 56.5″) in Nairobi, Kenya (Fig. 1). Donkey and horse ranged from 4–7 years old, however, ages of the wildlife species (zebra, waterbuck, buffalo, warthog) were unknown. Except for horses, individuals of each vertebrate species (five replicates) were sampled from different groups within the parks to capture possible diversity in skin volatile chemical profiles.

### Skin Odor Collection and Trapping

Skin odor samples were collected from five adult individuals of each vertebrate species. Zebra, waterbuck and buffalo were darted in the wild before skin odour collection, as described in Olaide et al. (2019). Immobilization drug composed of opiate etorphine hydrochloride (Captivon® 98, Wildlife Pharmaceuticals Ltd, South Africa) and azaperone (Kyron Laboratories, South Africa) (Olaide et al. 2019; Walzer 2007). The vertebrates were located using a vehicle and immobilization drug safely delivered in a 3 ml dart with a plain 50 mm needle shot using a Dan-Inject CO_2_ Injection rifle (Dan-Inject APS, Denmark). After skin odor collection, anaesthesia was reversed using intravenous injection of opioid antagonist diprenorphine (Activon®, Wildlife Pharmaceuticals Ltd, South Africa) at a dose of 0.045 mg/kg body weight (Olaide et al. 2019; Walzer 2007). All individual vertebrates recovered, with no complications observed after antagonist administration. Warthogs were captured using a nylon capture net, and were restrained manually by trained capture rangers prior to collection of skin odors.

Skin odor collection and trapping protocol is as described in Olaide et al. (2019) and Tchouassi et al. (2013). Briefly, Soxhlet-extracted cotton materials (23 cm × 23 cm, Lux Premium, Bidhannagar, West Bengal, India) were rubbed on each vertebrate’s skin around the belly and upper parts of the front legs which are major focus areas for tsetse feeding, for 10—12 min, using latex-gloved hands. For each replicate, headspace odors (volatile organic compounds, VOCs) were immediately collected onsite from the cotton materials trapped onto two pre-packed adsorbent filters (Carbopak B 3.5″ with 30 mg ± 5 mg, Sigma Scientific, Gainesville, Florida, US). Odor trapping from cotton materials onto adsorbent filters was carried out for 12 h using a portable field pump (USDA/CMAVE, Gainesville, Florida, USA), fitted with a push–pull headspace volatile trapping system supplying charcoal-filtered air at a flow rate of 348 ml/min. Tightly sealed (0.075 mm P.T.F.E. thread seal tape MAAT, UK) Carbopak B adsorbents with trapped headspace volatiles were wrapped in aluminum foil and transported to the laboratories at *icipe*, Nairobi in a cool box underlaid with carbon dioxide CO_2_ (dry ice pellets, Carbacid Investment Limited, Nairobi, Kenya). Trapped skin odors were eluted from each adsorbent filter under laboratory conditions with 200 μl gas chromatography (GC) grade dichloromethane into 2 ml glass vials, concentrated to 100 μl under a gentle stream of charcoal filtered nitrogen gas and kept at −80 °C for GC/MS analysis.

### Chemical Analysis

Skin odor samples were analyzed on a HP 7890 A series gas chromatograph (GC) tandem HP 5975 C mass spectrometer (MS) (Agilent Technologies, Wilmington, USA) fitted with an autosampler, a split-splitless injection port (200 ˚C), an Agilent HP-5MS non-polar capillary column (5% phenyl and 95% methylpolysiloxane, 30 m length × 250 μm i.d. × 0.25 μm film thickness), an Agilent technologies 5975C EIMS (70 eV) triple axis MS, and an Agilent ChemStation data system. The aliquot (1 μl) skin odor extracts were analyzed in splitless mode (purge flow to split vent 3 mL/min at 0.8 min) with temperature programming of the column oven (35 ˚C for 5 min then increase at 10 ˚C/min to 280 ˚C and held at this temperature for 10.5 min) and helium as the carrier gas (1.2 ml/min flow rate, 8.8271 psi head pressure). The GC injector and MS transfer line were kept at 270 ˚C and 280 ˚C, respectively, while the MS ion source and quadrupole were respectively kept at 230 ˚C and 150 ˚C. Mass spectra were acquired with a solvent delay of 3 min, with mass ranging from 38—550 Daltons (Da) and a threshold of 70 Da at a scan time of 0.73 scans/sec. The volatile organic compounds were tentatively identified using their retention times, electron ionization mass spectra and Kovats retention indices (RIs) which were compared with library GC/MS data (NIST11, Adams2 and Chemecol), published mass spectra and RIs from online NIST library database. Additionally, the identities of the previously identified repellent ketones 6-methyl-5-hepten-2-one, acetophenone, and geranyl acetone (Olaide et al. 2019) were confirmed with commercially purchased standards. The RIs of the VOCs were obtained using commercial standards of straight chain alkanes (C_7_-C_30_, 49,451-U, Supelco, Bellefonte, Pennsylvania, USA) run on the GC/MS using the same conditions described earlier for the skin odor samples. Retention indices (non-isothermal) were calculated using the formula: ${I}_{x}=100(n+({t}_{x}-{t}_{n})/({t}_{n+1}-{t}_{n})$, according to Van den Dool and Kratz (1963) for temperature-progammed GC where ${I}_{x}$ is the Kovats retention index of VOC $X$ with retention time ${t}_{x}$, ${t}_{n}$ and ${t}_{n+1}$ respectively are the retention times of the reference n-alkanes eluting immediately before and after the VOC $X$, and $n$ is the number of carbon atoms in the n-alkane eluting before VOC $X$. Using the absolute peak areas (abundance), the amount (μg/μl) of the repellent ketones were quantified in the skin odor samples of the vertebrates. To achieve this, external quantification was done using commercial samples of acetophenone for the benzenoid ketone and geranyl acetone for the aliphatic ketones prepared in five known concentrations (50 ng/µL to 250 ng/µL) within the expected range in the crude skin odors (Olaide et al. 2019). The peak areas of the commercial standards obtained after GC–MS analysis were used to generate calibration curves and linear equations (*R*^2^ = 0.986, *y* = 6*E* + 06*x* − 2*E* + 07 for acetophenone; and *R*^2^ = 0.988, *y* = 1*E* + 06*x* − 2*E* + 07 for geranyl acetone and 6-methyl-5-hepten-2-one) from which the naturally occurring-amounts in the skin odors were estimated. All peaks detected in the control (blank cotton material) were considered as contaminants and therefore discarded in the volatile analysis.

### Chemicals

The commercial chemicals used in this study were sourced as follows: 6-methyl-5-hepten-2-one (99%, M48805, Sigma-Aldrich), acetophenone (99%, A10701, Sigma-Aldrich), geranyl acetone (96%, 328,677, Sigma-Aldrich), dichloromethane solvent (≥ 99.9%, analytical grade, 270,997, Sigma-Aldrich) and C_7_-C_30_ straight chain alkanes (49,451-U, Supelco).

### Statistical Analyses

All data analyses of the skin-derived volatile organic compounds (VOCs) of zebra, donkey, horse, waterbuck, buffalo and warthog were performed using R statistical software version 4.4.1, in the R Studio graphical user interface (R Core Team 2024). The dataset (abundance of the VOCs across replicates of the different vertebrates) was first tested for normality using Shapiro–Wilk’s test, and homogeneity of variance using Bartlett’s test. Since the dataset was found to be not normally distributed and with non-homogenous variance, it was analyzed using the non-parametric tests Kruskal–Wallis test to compare abundance of the VOCs across the different vertebrates. When a significant difference was observed (P < 0.05), a Dunn’s post hoc test with Bonferroni adjustment was applied for means separation using the “dunn.test” package (Dinno 2024). The VOCs that were detected in only two groups (vertebrate species) were analyzed using the Mann–Whitney U test. All analyses were carried out at 5% probability level, i.e. *alpha* < 0.05. The machine learning algorithm random forest (RF) analysis (Breiman 2001) was employed to select the most discriminating VOCs among the vertebrates, based on the mean decrease in accuracy (MDA) obtained using the function “importance” in the “randomForest” package. The higher the MDA value, the higher its importance to discrimination (Liaw and Wiener 2002; Ranganathan and Borges 2010). Based on the abundance of top most discriminating VOCs, Sparse Partial Least Square Discriminant Analysis (sPLS-DA) was performed to visualize similarity of the vertebrate skin VOC profiles using the “mixOmics” and “ade4” packages (Dray and Dufour 2007; Hervé et al.^2018^; Lê Cao et al. 2011; Thioulouse et al 2018) as in previous studies (Adams et al. 2023; Ayelo et al. 2022). The function “tune.splsda” in the “mixOmics” package was used to select the optimum number of components and variables (volatiles) in the sPLS-DA model for enhanced classification accuracy. Further, biplot of the sPLS-DA was performed using the function “biplot” in the “mixOmics” package (Rohart et al. 2017), to illustrate variations in the VOC profiles of the different vertebrates and to highlight the correlation between the top discriminating VOCs and the different vertebrates. A clustering heatmap was carried out using the function “cim” in the “mixOmics” package (Rohart et al. 2017), to illustrate the variations in emission of the most discriminating VOCs across skin odors of replicate vertebrates. The out-of-bag error of the RF analysis was used to estimate the classification accuracy (1-OOB error) (Liaw and Wiener 2002; Ranganathan and Borges 2010). Similarly, the quality of the sPLS-DA model fit and prediction accuracy was validated using the function “perf”, the quality parameters R2X and R2Y (explaining the variation in the most discriminating VOCs and vertebrate groups, respectively), and the “leave-one-group-out” cross-validation method in the “mixOmics” package (Rohart et al. 2017). The sPLS-DA plot, biplot and clustering heatmap were also used to visualize and highlight the variations in the occurrence, abundance and natural ratios of previously identified repellent compounds across the different vertebrates.

### Ethics Statement

The Kenyan Wildlife Service KWS approved the sampling of skin odors of buffalo, warthog, waterbuck, and zebra (permit number: KWS/BRM/5001). Internal ethical clearance for use of donkey and horse was sought from *icipe’s* institutional biosafety committee. Further, oral consent from the horse and donkey farmers was sought to collect skin odor samples from their animals for use in our study.

## Results

### Analysis of Volatile Organic Compounds In Vertebrate Skin Odors

**Fig. 2:**
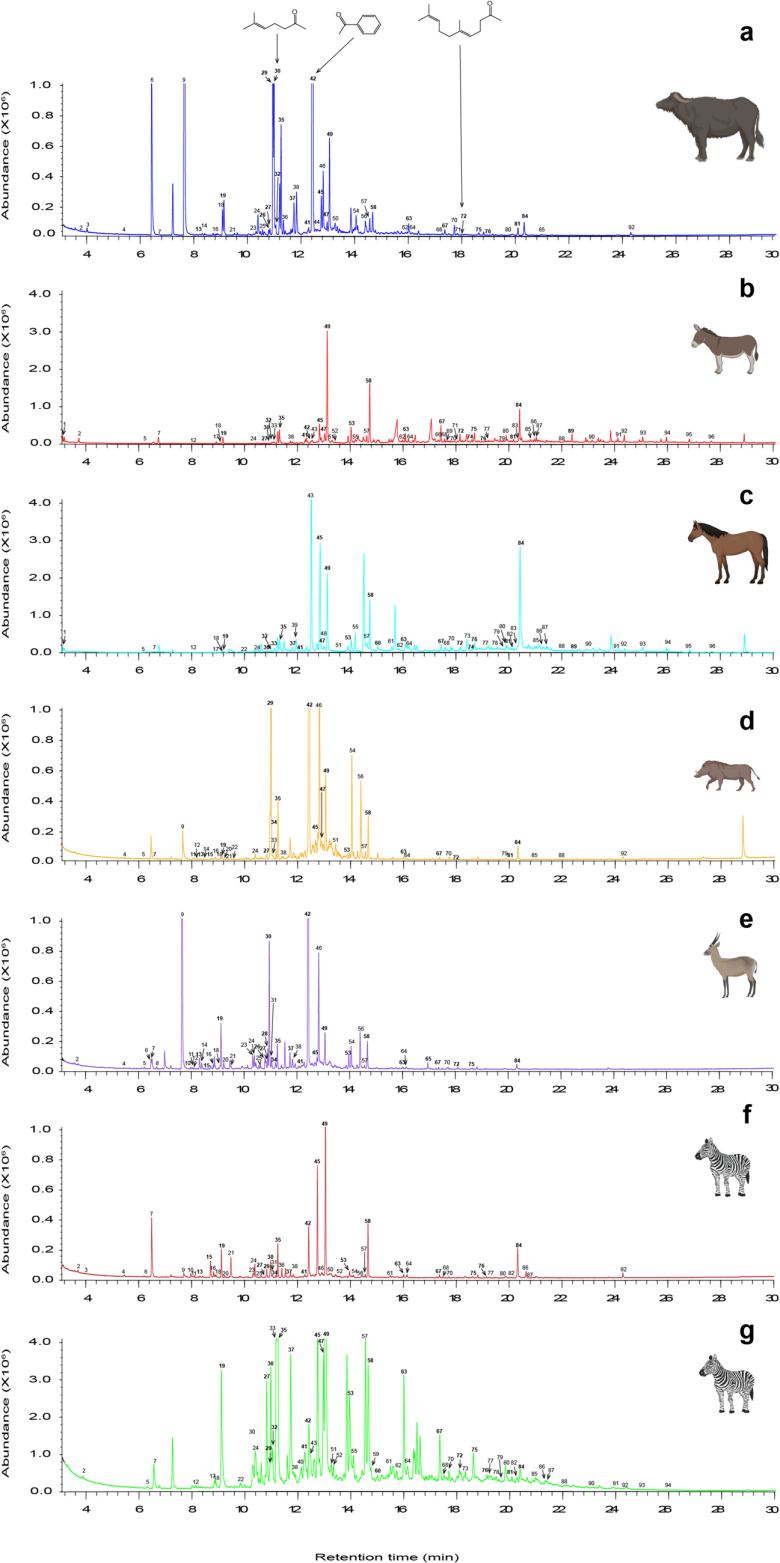
Representative total ion chromatograms (TIC) of the skin volatile emission profiles of different vertebrates. (**a**) buffalo, (**b**) donkey, (**c**) horse, (**d**) warthog, (**e**) waterbuck, (**g**) and (**h**) zebra skin odors collected in Amboseli and Nguruman, respectively. Numbers correspond to the list presented in Table 1. The most discriminating VOCs distinguishing the vertebrates (“mean decrease in accuracy” in random forest analysis) are in bold. Chemical structures of the repellent ketones previously identified from zebra skin odor, that is 6-methyl-5-hepten-2-one (**30**), acetophenone (**42**) and geranyl acetone (**72**), are shown. Vertebrate illustrations are not to scale, obtained via BioRender.com and Flaticon.com

A total of 96 volatile organic compounds (VOCs) were detected in the headspace skin emission profiles of buffalo, donkey, horse, warthog, waterbuck, and zebra (Fig. 2, Table 1). These compounds belong to 11 chemical classes namely: alkanes (20), alkenes (9), benzenoids (17), alcohols (10), phenols (2), furans (3), ketones (11), aldehydes (15), benzoquinone (1), esters (4), and a lactone (1), which vary among the vertebrate species. Alkanes ranged between (1.84–25.45%), alkenes (0.21–6.16%), benzenoids (1.17–9.67%), alcohols (6.57–29.94%), phenols (0–10.73%), furans (0–1.0%), ketones (4.81–59.14%), aldehydes (12.22–45.19%), benzoquinone (0–0.78%), esters (0.44–3.30%), and lactone (0–0.68%). The least and most abundant VOCs in the different classes were: alkanes (dodecane-15.38%, tetracosane-57.48%); alkenes ((*E*)-oct-2-ene-0.17%, dodec-1-ene-32.92%); benzenoids (toluene-0.25%, α-methylstyrene-23.89%); alcohols (hexanol-0.21%, α,α-dimethylbenzenemethanol-45.13%); phenols (p-cresol-44.44%, o-guaiacol-55.56%); furans (2,4-dimethylfuran-25.24%, 2-pentylfuran-47.92%); ketones (acetophenone- 45.14%, 2,3-octanedione-0.22%); aldehydes (nonanal- 39.48%, (*E*)-hex-2-enal-0.1%); and esters (hex-3-enyl acetate-4.03%, isopropyl hexadecanoate-59.48%).

**Table 1:** Volatile Organic Compounds Detected In The Skin Emission Profiles Of Preferred And Non-Preferred Vertebrate Hosts Of Tsetse Flies (n = 5)

							Median Abundance (Peak Area) (× 10^6^) ± SEMdn^g^							
Peak no.^a^	t_R_^b^ (min)	*I*^c^_Obs_	*I*^d^_Lit_	Compound^e^	MF^b^	Chemical class^f^	Buffalo	Donkey	Horse	Warthog	Waterbuck	Zebra_ANP	Zebra_Ngu	*P* value^h^
1	3.12	-	686^A^	pentan-2-one	C_5_H_10_O	ketone	nd	1.53 ± 0.17	1.10 ± 0.55	nd	nd	nd	nd	0.53
2	3.86	700	702^A^	pentanal	C_5_H_10_O	aldehyde	0.00 ± 0.34^a^	0.28 ± 0.13^ab^	nd	nd	0.22 ± 0.23^ab^	0.20 ± 0.11^b^	1.79 ± 0.80^a^	**0.023**
3	4.02	706	695^B^	2,4-dimethylfuran	C_6_H_8_O	furan	0.00 ± 0.15	nd	nd	nd	nd	0.18 ± 0.11	nd	1
4	5.46	760	762^A^	toluene	C_7_H_8_	benzenoid	0.00 ± 0.05^a^	nd	nd	0.30 ± 0.07^b^	0.16 ± 0.06^ab^	0.14 ± 0.05^ab^	nd	**0.019**
5	6.27	790	789.6^B^	oct-1-ene	C_8_H_16_	alkene	nd	0.21 ± 0.11	0.00 ± 0.10	0.00 ± 0.40	0.23 ± 0.17	nd	0.96 ± 0.65	0.369
6	6.44	797	782^C^	4-methyl-3-penten-2-one	C_6_H_10_O	ketone	26.06 ± 13.61	nd	nd	nd	1.00 ± 1.66	20.53 ± 12.13	nd	0.117
7	6.56	801	800^D^	hexanal	C_6_H_12_O	aldehyde	0.00 ± 0.24^b^	0.98 ± 0.18^ab^	0.58 ± 0.31^ab^	0.75 ± 0.40^ab^	4.83 ± 3.53^a^	0.00 ± 1.95^ab^	6.70 ± 5.61^a^	**0.014**
8	6.9	815	797.9^B^	(*E*)-oct-2-ene	C_8_H_16_	alkene	nd	nd	nd	nd	0.16 ± 0.06	nd	nd	
9	7.66	844	841.3^B^	4-hydroxy-4-methylpentan-2-one	C_6_H_12_O_2_	ketone	26.71 ± 12.89^a^	nd	nd	1.37 ± 5.72^b^	10.46 ± 10.50^ab^	4.89 ± 3.05^b^	nd	**0.021**
10	7.97	856	854^E^	(*E*)-hex-2-enal	C_6_H_10_O	aldehyde	nd	nd	nd	nd	0.52 ± 0.46	0.00 ± 0.19	nd	0.332
11	8.04	859	855^F^	2-furanmethanol	C_5_H_6_O_2_	furan	nd	nd	nd	0.19 ± 0.08^ab^	0.34 ± 0.08^a^	0.00 ± 0.06^b^	nd	**0.018**
12	8.13	863	868.3^B^	ethylbenzene	C_8_H_10_	benzenoid	nd	0.00 ± 0.09	0.00 ± 0.14	0.20 ± 0.12	0.18 ± 0.02	nd	1.48 ± 1.28	0.285
13	8.35	871	877.8^B^	1,3-dimethylbenzene^*^	C_8_H_10_	benzenoid	0.38 ± 0.25^ab^	nd	nd	0.24 ± 0.37^ab^	1.37 ± 0.70^a^	0.12 ± 0.05^b^	nd	**0.013**
14	8.41	874	871^G^	hexanol	C_6_H_14_O	alcohol	nd	nd	nd	0.26 ± 0.10	0.44 ± 0.15	nd	nd	0.105
15	8.74	887	884^F^	4-cyclopentene-1,3-dione^*^	C_5_H_4_O_2_	ketone	nd	nd	nd	0.31 ± 0.09	0.72 ± 0.23	0.43 ± 0.47	nd	0.151
16	8.84	891	890^D^	styrene	C_8_H_8_	benzenoid	0.14 ± 0.10	nd	nd	0.25 ± 0.08	0.36 ± 0.08	0.22 ± 0.17	nd	0.449
17	8.92	894	893^E^	non-1-ene	C_9_H_18_	alkene	nd	1.06 ± 0.32	0.00 ± 0.26	nd	nd	nd	0.00 ± 3.73	0.13
18	9.1	901	900^E^	nonane	C_9_H_2_0	alkane	1.11 ± 0.61^b^	0.58 ± 0.24^ab^	1.01 ± 0.17^ab^	0.27 ± 0.25^ab^	0.26 ± 0.13^a^	0.50 ± 0.15^ab^	0.00 ± 1.03^ab^	**0.048**
19	9.16	904	903^A^	heptanal^*^	C_7_H_14_O	aldehyde	4.21 ± 2.26^ab^	3.16 ± 6.21^ab^	1.68 ± 0.51^b^	1.36 ± 0.38^b^	5.01 ± 1.46^ab^	3.13 ± 0.88^ab^	94.03 ± 53.39^a^	** < 0.001**
20	9.23	907	909^H^	2-butoxyethanol	C_6_H_14_O_2_	alcohol	nd	nd	nd	0.00 ± 0.92	0.94 ± 0.33	0.39 ± 0.28	nd	0.785
21	9.5	920	888^I^	1,4-benzoquinone	C_6_H_4_O_2_	benzoquinone	0.00 ± 0.12	nd	nd	0.00 ± 0.70	0.51 ± 0.04	0.33 ± 0.61	nd	0.12
22	9.85	937	933.3^B^	2,6-dimethyloctane	C_10_H_22_	alkane	nd	nd	0.76 ± 0.32	0.00 ± 0.15	nd	nd	4.96 ± 1.42	0.061
23	10.34	960	956^D^	(*E*)-hept-2-enal	C_7_H_12_O	aldehyde	0.79 ± 0.67	nd	nd	nd	1.53 ± 0.30	0.34 ± 0.49	nd	0.539
24	10.4	963	962^A^	benzaldehyde	C_7_H_6_O	Aldehyde^#^	3.05 ± 1.10^ab^	0.80 ± 0.40^b^	3.07 ± 0.52^a^	1.36 ± 0.41^ab^	1.48 ± 0.20^ab^	1.71 ± 0.32^ab^	36.26 ± 19.55^a^	**0.016**
25	10.63	974	969^D^	heptanol	C_7_H_16_O	alcohol	0.41 ± 0.27	nd	nd	nd	0.36 ± 0.18	0.30 ± 0.19	nd	0.804
26	10.8	982		unidentified1^*^		-	0.68 ± 0.57	nd	nd	nd	0.71 ± 0.24	nd	nd	1
27	10.83	983	987.9^B^	α-methyl styrene^*^	C_9_H_10_	benzenoid	0.80 ± 0.33^ab^	0.00 ± 0.17^b^	nd	1.00 ± 0.19^ab^	0.97 ± 0.31^b^	1.17 ± 0.27^ab^	65.19 ± 32.48^a^	**0.002**
28	10.9	987	987^ J^	2,3-octanedione^*^	C_8_H_14_O_2_	ketone	nd	nd	nd	nd	1.23 ± 0.53	nd	nd	
29	10.99	991	997^ K^	2,2,4,6,6-pentamethylheptane^*^	C_12_H_26_	alkane	4.76 ± 4.84^b^	nd	nd	14.05 ± 7.60^ab^	nd	2.44 ± 4.00^b^	86.27 ± 12.55^a^	**0.004**
30	10.99	991	987^E^	6-methyl-5-hepten-2-one^*^	C_8_H_14_O	ketone	23.32 ± 6.84^a^	0.44 ± 0.35^b^	2.89 ± 0.72^ab^	nd	3.96 ± 2.65^ab^	5.74 ± 4.07^ab^	24.68 ± 13.39^a^	**0.003**
31	11.04	993	993^E^	2-pentylfuran	C_9_H_14_O	furan	nd	nd	nd	nd	0.91 ± 0.33	0.43 ± 0.20	nd	0.209
32	11.07	995	1000^B^	1,2,4-trimethylbenzene^*^	C_9_H_12_	benzenoid	1.07 ± 0.28	0.91 ± 1.53	0.00 ± 0.66	nd	nd	nd	4.17 ± 8.65	0.097
33	11.08	995	974.2^B^	mesitylene	C_9_H_12_	benzenoid	nd	0.47 ± 0.66	3.54 ± 1.39	0.58 ± 0.63	nd	nd	10.71 ± 8.58	0.082
34	11.19	1001	1000^L^	decane^*^	C_10_H_22_	alkane	nd	nd	nd	0.68 ± 0.60	0.62 ± 0.19	0.00 ± 0.25	nd	0.092
35	11.26	1004	1004^G^	octanal^*^	C_8_H_16_O	aldehyde	11.30 ± 3.36^ab^	8.38 ± 1.09^ab^	11.64 ± 2.37^ab^	4.28 ± 1.06^b^	1.63 ± 0.34^b^	3.71 ± 1.57^b^	177.80 ± 69.14^a^	** < 0.001**
36	11.35	1009	1004^ M^	hex-3-enyl acetate	C_8_H_14_O_2_	ester	0.56 ± 0.42	nd	nd	nd	nd	0.53 ± 0.27	nd	1
37	11.74	1031	1030^N^	2-ethylhexan-1-ol^*^	C_8_H_18_O	alcohol	2.39 ± 1.13	nd	0.38 ± 1.40	nd	0.90 ± 0.40	1.46 ± 0.34	52.12 ± 33.99	0.125
38	11.9	1040	1033^D^	benzyl alcohol	C_7_H_8_O	alcohol^#^	6.77 ± 7.91	1.43 ± 0.56	nd	1.05 ± 0.46	0.85 ± 0.26	2.41 ± 0.83	0.00 ± 3.97	0.157
39	11.95	1043		unidentified2		-	nd	nd	10.90 ± 3.04	nd	nd	nd	nd	
40	12.14	1054	1058.2^B^	1-methyl-3-propylbenzene	C_10_H_14_	benzenoid	nd	nd	nd	nd	nd	nd	7.67 ± 10.60	
41	12.28	1061	1058.2^B^	(*E*)-oct-2-enal^*^	C_8_H_14_O	aldehyde	3.75 ± 0.70	3.52 ± 0.59	2.42 ± 1.58	nd	1.17 ± 0.22	1.39 ± 0.51	0.00 ± 11.36	0.178
42	12.44	1070	1067^E^	acetophenone^*^	C_8_H_8_O	ketone^#^	109.93 ± 32.69^b^	2.79 ± 0.63^a^	nd	46.97 ± 34.56^ab^	22.66 ± 16.85^ab^	26.32 ± 23.85^ab^	47.44 ± 12.79^ab^	**0.022**
43	12.5	1074	1070^D^	octan-1-ol	C_8_H_18_O	alcohol	nd	2.29 ± 0.82^a^	93.86 ± 20.11^b^	nd	nd	nd	0.00 ± 10.40^a^	**0.016**
44	12.55	1077	1086^A^	p-cresol	C_7_H_8_O	phenol	1.55 ± 14.37	nd	nd	nd	nd	nd	nd	
45	12.76	1088	1089.6^B^	α,α-dimethylbenzenemethanol^*^	C_9_H_12_O	alcohol^#^	5.03 ± 1.47^b^	13.96 ± 3.60^ab^	69.54 ± 13.12^ab^	6.15 ± 3.31^b^	3.40 ± 3.61^b^	2.45 ± 4.56^b^	120.04 ± 7.78^a^	** < 0.001**
46	12.83	1092	1090^G^	o-guaiacol	C_7_H_8_O_2_	phenol	7.99 ± 1.83	nd	nd	5.43 ± 7.20	4.56 ± 3.23	2.21 ± 1.72	nd	0.508
47	12.98	1101	1100^B^	undecane^*^	C_11_H_24_	alkane	2.07 ± 1.04^b^	7.86 ± 2.35^ab^	0.00 ± 1.37^b^	2.04 ± 0.91^b^	nd	nd	107.42 ± 12.28^a^	**0.005**
48	13.01	1103	1100^B^	methylbenzoate	C_8_H_8_O_2_	ester^#^	nd	nd	4.84 ± 4.07	nd	nd	nd	nd	
49	13.07	1106	1103^D^	nonanal^*^	C_9_H_18_O	aldehyde	13.49 ± 2.73^bc^	53.88 ± 3.^26ab^	44.76 ± 2.72^ab^	10.64 ± 2.09^bc^	3.58 ± 0.77^c^	17.34 ± 5.35^abc^	447.65 ± 35.38^a^	** < 0.001**
50	13.23	1116	1259^O^	4-methylundecane	C_12_H_26_	alkane	1.87 ± 0.69	nd	nd	nd	nd	0.94 ± 1.18	nd	0.83
51	13.42	1128	1031^A^	p-cymene	C_10_H_14_	benzenoid^$^	nd	0.00 ± 0.86	0.00 ± 0.44	1.52 ± 0.81	nd	nd	11.43 ± 6.96	0.323
52	13.42	1128	1026^P^	o-cymene	C_10_H_14_	benzenoid^$^	nd	1.50 ± 0.51	nd	nd	nd	0.90 ± 1.05	24.27 ± 12.86	0.124
53	13.97	1163	1163^G^	(*E*)-non-2-enal^*^	C_9_H_16_O	aldehyde	nd	6.27 ± 1.32^abc^	9.32 ± 1.02^ab^	0.84 ± 0.35^bc^	1.14 ± 0.27^bc^	0.00 ± 0.44^c^	97.18 ± 30.32^a^	** < 0.001**
54	14.06	1169	1167^Q^	o-hydroxyacetophenone	C_8_H_8_O_2_	ketone*	3.78 ± 0.41	nd	nd	2.79 ± 2.61	1.42 ± 0.70	1.32 ± 0.82	nd	0.147
55	14.11	1172	1193^E^	dodec-1-ene	C_12_H_24_	alkene	nd	0.00 ± 0.58^b^	0.00 ± 0.90^b^	nd	nd	nd	23.22 ± 10.78^a^	**0.032**
56	14.4	1190	1181^D^	p-methylacetophenone	C_9_H_10_O	ketone^#^	2.56 ± 0.73	nd	nd	2.84 ± 2.04	1.68 ± 1.21	1.02 ± 0.79	nd	0.395
57	14.55	1199	1200^E^	dodecane	C_12_H_26_	alkane	1.44 ± 0.51^ab^	5.02 ± 1.67^ab^	8.12 ± 2.14^ab^	0.53 ± 0.54^b^	0.43 ± 0.17^b^	0.81 ± 0.17^b^	115.38 ± 14.91^a^	**0.003**
58	14.66	1207	1207^E^	decanal^*^	C_10_H_20_O	aldehyde	3.61 ± 0.44^bc^	19.21 ± 6.69^abc^	28.23 ± 2.85^ab^	6.22 ± 0.74^abc^	2.14 ± 0.52^c^	2.46 ± 1.22^bc^	108.67 ± 14.43^a^	** < 0.001**
59	14.78	1215	1213.6^L^	2,6-dimethylundecane	C_13_H_28_	alkane	nd	nd	nd	nd	nd	nd	7.89 ± 8.07	
60	15.04	1233		unidentified3^*^		-	nd	nd	8.03 ± 1.76	nd	nd	nd	0.00 ± 3.54	0.131
61	15.51	1266	1264^E^	(*E*)-dec-2-enal	C_10_H_18_O	aldehyde	nd	nd	5.38 ± 7.36	nd	nd	0.49 ± 0.24	0.00 ± 4.79	0.143
62	15.9	1292	1293^E^	tridec-1-ene	C_13_H_26_	alkene	0.55 ± 0.48	2.47 ± 0.81	0.00 ± 1.05	nd	nd	nd	0.00 ± 2.43	0.284
63	16.02	1301	1300^G^	tridecane^*^	C_13_H_28_	alkane	0.97 ± 0.34^abc^	6.66 ± 0.71^ab^	6.51 ± 1.39^ab^	0.54 ± 0.62^bc^	0.25 ± 0.05^c^	0.74 ± 0.12^bc^	87.51 ± 18.66^a^	** < 0.001**
64	16.16	1311	1308.8^B^	undecanal	C_11_H_22_O	aldehyde	0.00 ± 0.47^b^	3.35 ± 0.99^ab^	12.36 ± 5.73^a^	0.00 ± 0.21^b^	0.52 ± 0.09^ab^	0.62 ± 0.22^ab^	29.42 ± 11.82^ab^	**0.024**
65	16.96	1370	1260^D^	γ-octalactone^*^	C_8_H_14_O_2_	lactone	nd	nd	nd	nd	0.84 ± 0.19	nd	nd	
66	17.23	1390	1393^E^	tetradec-1-ene	C_14_H_28_	alkene	0.24 ± 0.09	2.12 ± 0.83	nd	nd	nd	nd	nd	0.389
67	17.36	1400	1400^N^	tetradecane^*^	C_14_H_30_	alkane	1.01 ± 0.71^abc^	9.69 ± 0.74^ab^	6.52 ± 1.87^ab^	0.58 ± 0.23^bc^	0.31 ± 0.05^c^	0.46 ± 0.28^bc^	50.26 ± 19.54^a^	** < 0.001**
68	17.54	1414	1412^G^	dodecanal	C_12_H_24_O	aldehyde	nd	2.72 ± 0.81	0.00 ± 10.66	nd	nd	0.00 ± 0.07	0.00 ± 8.26	0.592
69	17.59	1418	1511^D^	tridecanal	C_13_H_26_O	aldehyde	nd	0.00 ± 1.08	3.38 ± 1.18	nd	nd	nd	nd	0.373
70	17.74	1430	1419^E^	α-cedrene	C_15_H_24_	alkene^$^	1.10 ± 0.32^ab^	4.14 ± 0.98^a^	3.08 ± 1.33^a^	0.37 ± 0.87^ab^	0.17 ± 0.07^b^	0.23 ± 0.19^ab^	15.82 ± 6.62^a^	**0.003**
71	17.88	1441	1424^G^	β-cedrene	C_15_H_24_	alkene^$^	0.00 ± 0.07	0.00 ± 0.62	nd	nd	nd	nd	nd	0.724
72	18.12	1459	1453^D^	geranyl acetone^*^	C_13_H_22_O	ketone^$^	0.00 ± 0.05^c^	4.60 ± 0.49^ab^	5.55 ± 0.66^ab^	0.00 ± 0.31^bc^	0.33 ± 0.07^abc^	nd	17.74 ± 8.08^a^	** < 0.001**
73	18.42	1483	1575^R^	tridecan-1-ol	C_13_H_28_O	alcohol	nd	nd	6.23 ± 6.75	nd	nd	nd	0.00 ± 1.71	0.119
74	18.61	1498	1772^S^	pentadecan-1-ol^*^	C_15_H_32_O	alcohol	nd	3.65 ± 0.30^a^	0.00 ± 0.73^b^	nd	nd	nd	nd	**0.009**
75	18.65	1501	1500^L^	pentadecane^*^	C_15_H_32_	alkane	0.33 ± 0.14^c^	7.42 ± 0.52^b^	7.03 ± 2.65^b^	nd	0.21 ± 0.04^c^	0.28 ± 0.12^c^	42.93 ± 17.70^a^	**0.018**
76	19.17	1544	1526^ T^	1-butylhexylbenzene^*^	C_16_H_26_	benzenoid	0.22 ± 0.40^c^	2.03 ± 0.71^b^	7.10 ± 3.04^a^	nd	nd	0.23 ± 0.21^c^	7.11 ± 5.92^a^	**0.025**
77	19.29	1554	1534^ T^	1-propylheptylbenzene	C_16_H_26_	benzenoid	nd	0.00 ± 0.75	2.23 ± 1.54	nd	nd	0.00 ± 0.04	0.00 ± 0.73	0.375
78	19.54	1575	1553^ T^	1-ethyloctylbenzene	C_16_H_26_	benzenoid	nd	nd	2.60 ± 1.22	nd	nd	nd	0.00 ± 1.52	0.451
79	19.84	1600	1587^U^	hexadec-1-ene	C_16_H_32_	alkene	nd	0.00 ± 0.67	0.00 ± 0.50	0.00 ± 0.23	nd	nd	0.00 ± 0.74	0.963
80	19.85	1601	1600^E^	hexadecane	C_16_H_34_	alkane	0.00 ± 0.40^b^	4.58 ± 0.45^ab^	2.65 ± 1.30^ab^	nd	nd	0.00 ± 0.19^b^	37.75 ± 19.09^a^	**0.005**
81	20.14	1627	1607.9^L^	cedrol^*^	C_15_H_26_O	Alcohol^$^	0.97 ± 0.36	3.88 ± 0.64	0.00 ± 1.13	0.00 ± 0.47	nd	nd	13.06 ± 6.16	0.072
82	20.2	1632	1626^ T^	1-butylheptylbenzene	C_17_H_28_	benzenoid	nd	nd	0.00 ± 0.35	nd	nd	0.00 ± 0.05	5.15 ± 5.24	0.165
83	20.34	1644	1620^ T^	1-pentylhexylbenzene	C_17_H_28_	benzenoid	nd	0.00 ± 0.40	1.84 ± 0.82	nd	nd	nd	nd	0.076
84	20.37	1647	1625^G^	benzophenone^*^	C_13_H_10_O	ketone*	0.61 ± 0.62^b^	13.42 ± 2.01^ab^	58.19 ± 16.13^a^	1.03 ± 0.55^b^	1.06 ± 0.57^b^	0.37 ± 1.09^b^	2.78 ± 7.97^ab^	**0.003**
85	21.01	1704	1700^L^	heptadecane	C_17_H_36_	alkane	0.00 ± 0.05	3.07 ± 0.94	2.23 ± 1.25	0.00 ± 0.19	nd	nd	3.80 ± 3.27	0.194
86	21.38	1738	1719^ T^	1-pentylheptylbenzene	C_18_H_30_	benzenoid	nd	2.37 ± 0.24	2.15 ± 1.03	nd	nd	0.00 ± 0.06	3.36 ± 4.67	0.050
87	21.44	1743	1723^ T^	1-butyloctylbenzene	C_18_H_30_	benzenoid	nd	0.00 ± 0.60	2.30 ± 1.49	nd	nd	0.00 ± 0.05	0.00 ± 2.26	0.194
88	22.08	1802	1800^E^	octadecane	C_18_H_38_	alkane	nd	2.05 ± 0.27	0.77 ± 0.88	0.00 ± 0.13	nd	nd	0.00 ± 3.83	0.059
89	22.4	1833	1831^ V^	isopropyl tetradecanoate^*^	C_17_H_34_O_2_	ester	nd	3.85 ± 0.56^a^	0.00 ± 0.40^b^	nd	nd	nd	nd	**0.01**
90	23.15	1906	1900^E^	nonadecane	C_19_H_40_	alkane	nd	3.02 ± 0.34	2.48 ± 2.23	nd	nd	nd	0.00 ± 11.35	0.127
91	24.08	2001	2000^E^	eicosane	C_20_H_42_	alkane	nd	3.01 ± 0.37	2.18 ± 0.86	nd	nd	nd	0.00 ± 1.79	0.455
92	24.37	2032	1999^D^	isopropyl hexadecanoate	C_19_H_38_O_2_	ester	0.16 ± 0.15	4.31 ± 0.58	2.78 ± 1.27	1.12 ± 0.39	nd	0.38 ± 0.38	8.83 ± 5.17	0.071
93	25.08	2108	2100^Q^	heneicosane	C_21_H_44_	alkane	nd	3.85 ± 1.08	4.20 ± 1.32	nd	nd	nd	0.00 ± 2.21	0.363
94	25.97	2207	2200^Q^	docosane	C_22_H_46_	alkane	nd	3.19 ± 0.30	2.74 ± 1.82	nd	nd	nd	0.00 ± 2.60	0.305
95	26.84	2307	2300^Q^	tricosane	C_23_H_48_	alkane	nd	2.23 ± 0.24	4.06 ± 1.79	nd	nd	nd	nd	0.143
96	27.67	2408	2400^Q^	tetracosane	C_24_H_50_	alkane	nd	1.47 ± 0.69	0.00 ± 1.16	nd	nd	nd	nd	0.656

^a^ Peak number correlates with numbering on the total ion chromatograms in Fig. 2

^b^ t_R_ (min) = retention time in minutes; MF = molecular formular

^c^ *I*_Obs_ = retention index observed in this study calculated based on C_7_-C_30_ n-alkanes of HP-5MS capillary column (30 m/0.25 mm/0.25 μm, He, 35 ˚C @ 5 min, 10 ˚C/min, 280 ˚C @ 10.5 min; T_end_: 280 ˚C)

^d^ *I*_Lit_ = retention index obtained from literature. References: ^A^ Bonaiti et al. 2005; ^B^ Xu et al. 2003; ^C^ Yamaguchi and Sibamoto-1981; ^D^ Pino et al. 2005; ^E^ Flamini et al. 2006; ^F^ Deport et al. 2006; ^G^ Asuming et al. 2005; ^H^ Cajka et al. 2007; ^I^ Zenkevich 2005; ^J^ Turchini et al. 2004; ^K^ Insausti et al. 2005; ^L^ Zeng et al. 2007; ^M^ Campeol et al. 2003; ^N^ Sartin et al. 2001; ^O^ Kotowska et al. 2012; ^P^ Vagionas et al. 2007; ^Q^ Radulovic et al. 2009; ^R^ Skaltsa et al. 2001; ^S^ Saroglou et al. 2007; ^T^ Peng et al. 1992; ^U^ Mimica-Dukic et al. 2003; ^V^Lalel et al. 2003

^e^ Compounds were identified based on their retention times, electron ionization mass spectra and retention indices compared with library GC/MS data (NIST11, Adams2 and Chemecol) and published mass spectra and retention indices s from online NIST library database. ^*^ indicates most discriminating VOCs distinguishing the vertebrates based on the “mean decrease in accuracy” in random forest analysis; compounds in bold are the primary focus repellent ketones in this study previously identified from zebra skin odor and for which identities were confirmed using authentic standards

^f^ Chemical class: compounds are also aromatic (^#^) or terpenoids (^$^)

^g^ Median Abundance (× 10^6^) ± standard error of the median (SEMdn): volatiles were collected from five biological replicates for each vertebrate species. Zebra_ANP and Zebra_Ngu = Zebra skin odor collected in Amboseli NP and Nguruman, respectively. Means with different superscript letters are significantly different (Kruskal-Wallis test, or Mann-Whitney U test where applicable, followed by Dunn’s post hoc test; P < 0.05). nd = not detected

^h^ *P* – value = probability value of the non-parametric Kruskal–Wallis and Mann–Whitney U tests comparing abundance of the 96 VOCs across the different vertebrates. Significant values (i.e. P < 0.05) are in bold

### Proposed Repellent Biomarkers for Less Preferred Vertebrate Hosts

The proposed three ketone repellent biomarkers 6-methyl-5-hepten-2-one, acetophenone and geranyl acetone previously identified from zebra skin odor for tsetse flies were generally present in all the vertebrates tested but were emitted at different levels (Kruskal–Wallis followed by Dunn’s test; *P* < 0.05; Table 1). Remarkably, all three ketones were detected in the non-preferred hosts waterbuck and zebra which was generally not the case for the preferred hosts buffalo, donkey, horse, and warthog. However, exceptions were recorded in the non-preferred zebra skin odor from Amboseli, where geranyl acetone was not detected. Further quantification of these compounds revealed their natural ratios in skin volatile emissions of the different vertebrate hosts (Table 2). Notably, where present, the ratios of acetophenone relative to the other two ketones were different for the non-preferred waterbuck and zebra compared to buffalo, donkey, horse, and warthog which are preferred hosts of tsetse flies.

**Table 2:** Ratios Of The Zebra-Derived Tsetse Repellent Ketones Across Different Vertebrate Skin Odors (n = 5)

Compound	buffalo	donkey	horse	waterbuck	warthog	zebra_ANP^a^	zebra_Ngu^a^
6-methyl-5-hepten-6-one	1	1	1	1	nd	1	1
acetophenone	4	1	nd	2	3	2	2
geranyl acetone	1^b^	1	1	1	1	nd	1

^a^ zebra_ANP and zebra_Ngu are skin odors collected from zebra in Amboseli NP and Nguruman, respectively

nd = not detected

^b^ detected in the skin emissions of only one individual buffalo

**Fig. 3:**
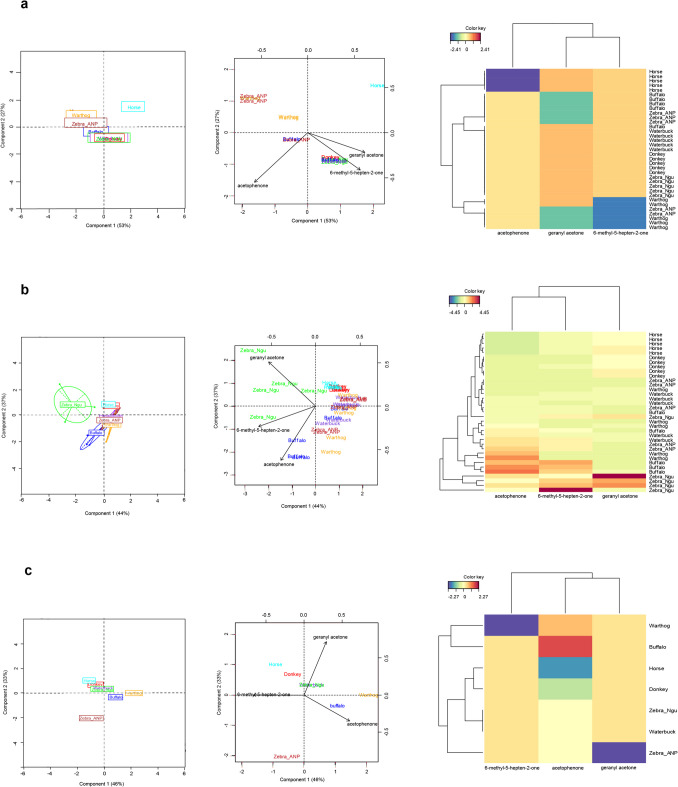
Variations in zebra skin-derived tsetse repellent ketones in non-preferred and preferred vertebrate hosts. Sparse partial least square discriminant analysis (sPLS-DA) grouping of the vertebrates (**left**), a sPLS-DA biplot classification of vertebrates and correlation of volatiles (**center**), and Heatmap clustering showing the abundance (in decreasing color intensity) across vertebrate skin odors (**right**) using (**a**) occurrence, (**b**) abundance and (**c**) natural ratios of occurrence of the repellent ketones 6-methyl-5-hepten-2-one, acetophenone and geranyl acetone found in the skin volatile emission profiles of different vertebrates. Zeb_ANP and Zebra_Ngu = zebra skin odor collected from Amboseli and Nguruman, respectively. Skin odors were collected from five (5) individuals for each vertebrate species

Based on the occurrence and abundance of the three ketones in the skin volatile emission profiles, the vertebrates were grouped together, except for horse (Fig. 3a) and zebra (Nguruman) (Fig. 3b), respectively. By contrast, using the ratios of these ketones the vertebrates were grouped into four: one composed of buffalo; a second group had only warthog; and a third group comprised zebra (Nguruman), waterbuck, donkey and horse (Fig. 3c). The latter group clustered closely together; and a fourth group had only zebra (Amboseli) which grouped separately from the others (Fig. 3c). The first two dimensions of the sPLS-DA accounted for 79% of the total variation with dimension 1 explaining 46% and dimension 2 accounting for 33% of the total variation. In addition, the clustering heatmap (horizontal direction) classified the vertebrates into two main groups, that is, warthog and buffalo, and others (Fig. 3c).

### Contribution of Background Skin Odors to Tsetse Fly Discrimination of Non-Preferred Vertebrates

**Fig. 4:**
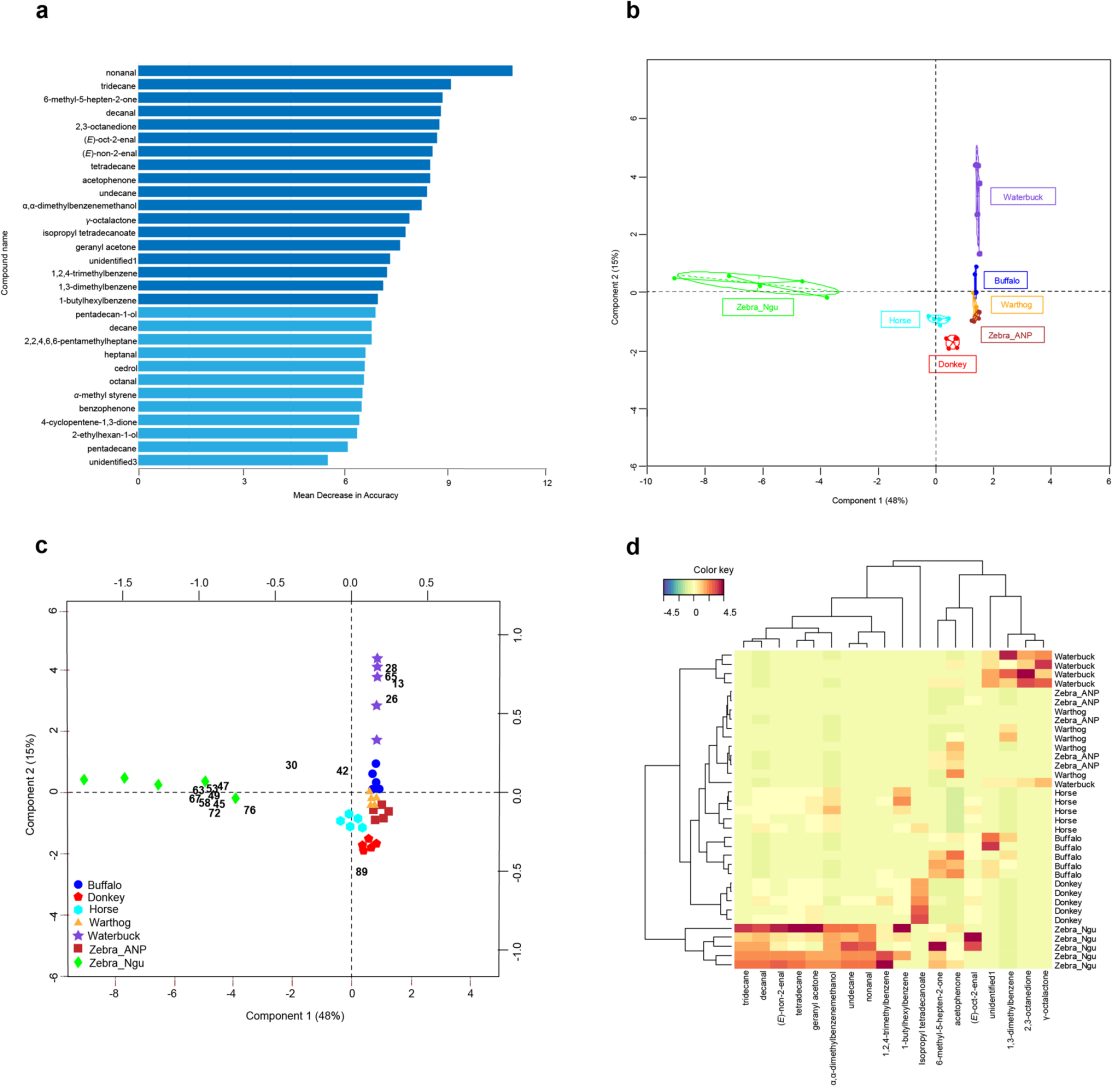
Discriminating volatile organic compounds (VOCs) and their correlation with different vertebrates. (**a**) Thirty (30) most discriminating VOCs in skin volatile emission profiles of buffalo, donkey, horse, warthog, waterbuck and zebra listed in decreasing importance based on the mean decrease in accuracy in the random forest analysis; **(b)** A sparse partial least square discriminant analysis (sPLS-DA) plot showing the distribution of VOCs across the vertebrate skin emissions using the top most discriminating volatiles (MDA > 7) (R2X = 0.81, R2Y = 0.82); **(c)** A sPLS-DA biplot showing the correlation of the top most discriminating VOCs with different vertebrates (R2X = 0.81, R2Y = 0.82). Numbers in bold within the plot are the discriminating VOCs corresponding to the numbering presented in Table 1 and total ion chromatograms in Fig. 2; **(d)** Heatmap clustering showing the abundance (in decreasing color intensity) of the most discriminant VOCs across replicate skin odors of the vertebrates. zebra_ANP = zebra skin odor collected from Amboseli; zebra_Ngu = zebra skin odor collected from Nguruman

Thirty (30) VOCs were found to be the best to distinguish between the different vertebrates based on the “mean decrease in accuracy” (Random Forest analysis, MDA) (Figs. 3a, 4). The abundance of which varied significantly across the vertebrates (Kruskal–Wallis test followed by Dunn’s post hoc test, or Mann–Whitney U test where applicable; *P* < 0.05) (Table 1). Using the top most discriminating compounds (MDA > 7), the vertebrates were grouped into four clusters comprising of: (i) zebra (Nguruman), (ii) waterbuck, (iii) buffalo, warthog, zebra (Amboseli), and horse, and (iv) donkey (Fig. 4b). While groups (i) and (ii) clustered completely separately, groups (iii) to (iv) clustered closely together. The quality parameters of the random forest (that is, 1—OOB error (out-of-bag error) of 97.14%) and sPLS-DA (i.e., R2X 0.81 and R2Y 0.82) models show their strong predictive power and accurate classification of the different vertebrates based on their specific skin-derived VOCs.

We found a correlation between the top discriminating VOCs and the vertebrates, most of which were associated with zebra (Nguruman) and waterbuck (Fig. 4c). The first two (2) dimensions of the sPLS-DA explained 63% of the total variation, with dimensions 1 and 2 contributing 48% and 15%, respectively. Dimension 1 was associated mainly with 1-butylhexylbenzene, 1,2,4-trimethylbenzene, geranyl acetone, tetradecane, (*Z*)-non-2-enal, α,α-dimethylbenzenemethanol, tridecane, undecane, decanal and nonanal correlated with zebra (Nguruman) (Fig. 4c). However, dimension 2 was primarily associated with undecane, geranyl acetone, 1-butylhexylbenzene and 6-methyl-5-hepten-2-one correlated with zebra (Nguruman), acetophenone correlated with buffalo, isopropyl tetradecanoate correlated with donkey, and 1,3-dimethylbenzene, 2,3-octanedione, γ-octalactone correlated with waterbuck (Fig. 4c). Clustering heatmap classified the vertebrates into two main categories; one group composed of zebra (Nguruman), and a second group made up of the remaining vertebrates which were subsequently grouped separately (Fig. 4d).

## Discussion

We found that the three repellent ketones 6-methyl-5-hepten-2-one, acetophenone and geranyl acetone previously identified in zebra skin odor were generally present in all the vertebrates tested, including buffalo, donkey, horse, warthog, waterbuck, and zebra. However, their ratios within the vertebrates varied specifically with acetophenone which appeared to distinguish non-preferred (zebra and waterbuck) and preferred (donkey, horse, buffalo, warthog) hosts. Additionally, our results suggest that background odors in each vertebrate’s skin volatile emission profiles could contribute to their overall discrimination by tsetse flies. Surprisingly, zebra skin odors collected in Nguruman and Amboseli National Park differed in their volatile emission profiles which could be associated with the sites. These results suggest that the three repellent ketones present in specific ratios combined with background vertebrate skin volatiles may serve as biomarkers for tsetse flies to distinguish between non-preferred- and preferred vertebrate hosts in their natural habitats.

In the current study, we found that while 6-methyl-5-hepten-2-one and acetophenone were common in zebra skin odors regardless of geographic location, geranyl acetone was detected only in Nguruman and not Amboseli suggesting it could be associated with genetic, physiological, pathological, and environmental factors (De Moraes et al. 2018; Emami et al. 2017; Stanczyk et al. 2018), which would require additional research.

Our analysis identified acetophenone as a potential discriminatory biomarker for tsetse flies to distinguish non-preferred and preferred hosts. Apart from occurrence (i.e. presence or absence) and concentration (i.e. abundance) in the skin volatile emissions, ratios in which individual compounds are found in skin odors could determine behavioral activity on arthropod vectors (Beyaert et al. 2010; Cha et al. 2011; Guerenstein and Lazzari 2009). Similarly, interactions of these compounds could dictate their effectiveness, as previously observed for phenols in cattle urine (Bursell et al. 1988; Vale et al. 1988). In turn, the ratios of behaviorally active repellents and their interactions could dictate the repellency of vertebrates which is consistent with our findings. Although this would require additional behavioral experiments to establish preferences of tsetse flies towards blends simulating the natural ratio of occurrence in the non-preferred and preferred vertebrates observed in this study. The role individual chemicals play in the repellency and their interaction with tsetse olfactory architecture vary (Diallo et al. 2020; Orubuloye et al. 2024). Previous research highlighted the importance of geranyl acetone as a superior spatial repellent compared to acetophenone (Olaide et al. 2021), and with strong antifeedant activity correlating with its effect on mRNA transcripts of the antennal olfactory receptors (ORs) of tsetse flies (Diallo et al. 2020). The higher vapor pressure of acetophenone than geranyl acetone could explain differences in their spatial repellency (Olaide et al. 2021). However, the antifeedant activity of acetophenone and their effects on mRNA transcripts of tsetse fly antennal ORs relative to geranyl acetone is currently unknown, and investigating this may unravel its importance in tsetse fly ecology.

Machine learning algorithms, such as random forest and sPLS-DA are increasingly applied in chemical ecology to identify discriminating VOCs involved in insect communication (Adams et al. 2023; Ayelo et al. 2022; Baleba et al. 2019; Hervé et al. 2018; Miano et al. 2022; Ranganathan and Borges 2010). This enhances the ability to handle complex data sets like the skin volatile emission profiles of vertebrates, identify patterns, and predict ecologically relevant components in the complex skin odor matrix. Interestingly, 6-methyl-5-hepten-2-one, acetophenone and geranyl acetone were among the most discriminating compounds of the 96 skin-derived VOCs detected in our study. Confirmation of the behavioral effects of other discriminatory compounds are required including assessment of physiological activity via electrophysiology.

We highlight three key limitations in our study. First, the sample size of individual vertebrate species used for odor collection was small (range) attributed to constraints in trapping wildlife and the use of vertebrates for research (Paul et al. 2016; Slijkerman et al. 2015; Verderio et al. 2023). Secondly, skin volatile emissions of vertebrates can vary by age and sex even for specific species as demonstrated in cattle (Torr et al. 2006, 2007), possibly linked to differing physiological states of the vertebrates and ecological roles of VOCs (Alberts 1992; Apps et al. 2015). Lastly, other potential confounders like host infection status, can contribute to the observed variation in the volatiles of the hosts examined (De Moraes et al. 2018; Emami et al. 2017; Stanczyk et al. 2018). However, this study provides valuable insights into the skin volatile emission profiles of vertebrate hosts and potential adaptive significance relevant to tsetse fly ecology.

We conclude that in nature, tsetse fly vertebrate hosts skin-derived volatiles vary based on the ecological settings, and the role of the three repellent ketones in discriminating non-preferred and preferred vertebrate hosts would depend on the skin background odor. As such geography, seasonality and a larger pool of vertebrates and sample size should be considered in investigating potential biomarkers that the vector may use to discriminate hosts for feeding. Additionally, the role of other sensory cues derived from physical sources including visual, thermal, tactile and acoustic combined with olfactory biomarkers should be investigated in future behavioral research for the integrated management of tsetse flies and African trypanosomosis.

## Data Availability

The original dataset generated during this study is available from the corresponding authors upon reasonable request.

## References

[CR1] Abro Z, Fetene GM, Kassie M, Melesse TM (2023) Socioeconomic burden of trypanosomiasis: evidence from crop and livestock production in Ethiopia. J Agric Econ 74(3):785–799. 10.1111/1477-9552.12531

[CR2] Adams B, Yusuf AA, Torto B, Khamis FM (2023) Non-host plant odors influence the tritrophic interaction between tomato, its foliar herbivore *Tuta absoluta* and mirid predator *Nesidiocoris tenuis*. Front Plant Sci 14:1014865. 10.3389/fpls.2023.1014865 37035056 10.3389/fpls.2023.1014865 PMC10076674

[CR3] Alberts AC (1992) Constraints on the design of chemical communication systems in terrestrial vertebrates. Am Nat 139:S62-89. 10.1086/285305

[CR4] Apps PJ, Weldon PJ, Kramer M (2015) Chemical signals in terrestrial vertebrates: search for design features. Nat Prod Rep 32(7):1131–1153. 10.1039/C5NP00029G 26100000 10.1039/c5np00029g

[CR5] Asuming WA, Beauchamp PS, Descalzo JT, Dev BC, Dev V, Frost S, Ma CW (2005) Essential oil composition of four Lomatium Raf species and their chemotaxonomy. Biochem Syst Ecol 33(1):17–26. 10.1016/j.bse.2004.06.005

[CR6] Auty H, Cleaveland S, Malele I, Masoy J, Lembo T, Bessell P, Torr S, Picozzi K, Welburn SC (2016) Quantifying heterogeneity in host-vector contact: tsetse (Glossina Swynnertoni and G. pallidi-pes) host choice in Serengeti National Park Tanzania. PLoS ONE 11(10):e0161291. 10.1371/journal.pone.0161291 27706167 10.1371/journal.pone.0161291 PMC5051720

[CR7] Ayelo PM, Mohamed SA, Chailleux A, Yusuf AA, Pirk CW, Deletre E (2022) The parasitoid *Dolichogenidea gelechiidivoris* eavesdrops on semiochemicals from its host *Tuta absoluta* and tomato. J Pest Sci 95(2):633–652. 10.1007/s10340-021-01424-w

[CR8] Baleba SB, Torto B, Masiga D, Weldon CW, Getahun MN (2019) Egg-laying decisions based on olfactory cues enhance offspring fitness in Stomoxys calcitrans L. (Diptera: Muscidae). Sci Rep 9(1):3850. 10.1038/s41598-019-40479-9 30846772 10.1038/s41598-019-40479-9 PMC6405918

[CR9] Bett MK, Saini RK, Hassanali A (2015) Repellency of tsetse-refractory waterbuck (*Kobus Defassa*) body odour to *Glossina pallidi-pes* (Diptera: Glossinidae): assessment of relative contribution of different classes and individual constituents. Acta Trop 146:17–24. 10.1016/j.actatropica.2015.02.017 25746973 10.1016/j.actatropica.2015.02.017

[CR10] Beyaert I, Waschke N, Scholz A, Varama M, Reinecke A, Hilker M (2010) Relevance of resource-indicating key volatiles and habitat odour for insect orientation. Anim Behav 79(5):1077–1086. 10.1016/j.anbehav.2010.02.001

[CR11] Bonaiti C, Irlinger F, Spinnler HE, Engel E (2005) An iterative sensory procedure to select odor-active associations in complex consortia of microorganisms: application to the construction of a cheese model. J Dairy Sci 88(5):1671–1684. 10.3168/jds.S0022-0302(05)72839-3 15829658 10.3168/jds.S0022-0302(05)72839-3

[CR12] Breiman L (2001) Random forests. Mach Learn 45:5–32. 10.1023/A:1010933404324

[CR13] Bursell E, Gough A, Beevor P, Cork A, Hall D, Vale G (1988) Identification of components of cattle urine attractive to tsetse flies, Glossina spp. (Diptera: Glossinidae). Bull Entomol Res 78:281–291. 10.1017/S0007485300013043

[CR14] Čajka T, Hajšlová J, Cochran J, Holadová K, Klimánková E (2007) Solid phase microextraction–comprehensive two-dimensional gas chromatography–time-of-flight mass spectrometry for the analysis of honey volatiles. J Sep Sci 30(4):534–546. 10.1002/jssc.200600413 17444222 10.1002/jssc.200600413

[CR15] Campeol E, Flamini G, Cioni PL, Morelli I, Cremonini R, Ceccarini L (2003) Volatile fractions from three cultivars of Olea eruopaea L. collected in two different seasons. J Agric Food Chem 51(7):1994–1999. 10.1021/jf026025u 12643664 10.1021/jf026025u

[CR16] Cha DH, Linn CE, Teal PEA, Zhang A, Roelofs WL, Loeb GM (2011) Eavesdropping on plant volatiles by a specialist moth: significance of ratio and concentration. PLoS ONE 6(2):e17033. 10.1371/journal.pone.0017033 21347337 10.1371/journal.pone.0017033 PMC3036738

[CR17] Channumsin M, Ciosi M, Masiga D, Auty H, Turner CM, Kilbride E, Mable BK (2021) Blood meal analysis of tsetse flies (*Glossina pallidipes*: Glossinidae) reveals higher host fidelity on wild com-pared with domestic hosts. Wellcome Open Res 6(213). 10.12688/wellcomeopenres.16978.1 10.12688/wellcomeopenres.16978.1 PMC8513123 34703903

[CR18] Clausen PH, Adeyemi I, Bauer B, Breloeer M, Salchow F, Staak C (1998) Host preferences of tsetse (Diptera: Glossinidae) based on bloodmeal identifications. Med Vet Entomol 12(2):169–180. 10.1046/j.1365-2915.1998.00097.x 9622371 10.1046/j.1365-2915.1998.00097.x

[CR19] De Moraes CM, Wanjiku C, Stanczyk NM, Pulido H, Sims JW, Betz HS, Read AF, Torto B, Mescher MC (2018) Volatile biomarkers of symptomatic and asymptomatic malaria infection in humans. Proc Natl Acad Sci 115(22):5780–5785. 10.1073/pnas.1801512115 29760095 10.1073/pnas.1801512115 PMC5984526

[CR20] Deport C, Ratel J, Berdagué J-L, Engel E (2006) Comprehensive combinatory standard correction: A calibration method for handling instrumental drifts of gas chromatography-mass spectrometry systems. J Chromatogr A 1116(1–2):248–258. 10.1016/j.chroma.2006.03.092 16631179 10.1016/j.chroma.2006.03.092

[CR21] Diallo S, Shahbaaz M, Torto B, Christoffels A, Masiga D, Getahun MN (2020) Cellular and molecular targets of waterbuck repel-lent blend odors in antennae of *Glossina fuscipes fuscipes* New-stead, 1910. Front Cell Neurosci 14:137. 10.3389/fncel.2020.00137 32581714 10.3389/fncel.2020.00137 PMC7283967

[CR22] Dinno A (2024) Dunn.test: dunn's test of multiple comparisons using rank sums. R package version 1.3.6. https://CRAN.R-project.org/package=dunn.test. Accessed 15 Sept 2024

[CR23] Dray S, Dufour A (2007) The ade4 Package: Implementing the Duality Diagram for Ecologists. J Stat Softw 22(4):1–20. 10.18637/jss.v022.i04

[CR24] Ebhodaghe FI, Okal MN, Kalayou S, Bastos AD, Masiga DK (2021) Tsetse bloodmeal analyses incriminate the common warthog *Phacochoerus africanus* as an important cryptic host of ani-mal trypanosomes in smallholder cattle farming communities in Shimba Hills. Kenya Pathogens 10(11):1501. 10.3390/pathogens10111501 34832656 10.3390/pathogens10111501 PMC8623152

[CR25] Emami SN, Lindberg BG, Hua S, Hill SR, Mozuraitis R, Lehmann P (2017) A key malaria metabolite modulates vector blood seeking, feeding, and susceptibility to infection. Science 355(6329):1076–1080. 10.1126/science.aah4563 28183997 10.1126/science.aah4563

[CR26] FAO and WHO (2022) Vector control and the elimination of gam-biense human African trypanosomiasis (HAT). Joint FAO/WHO Virtual Expert Meeting 5–6 Oct 2021 PAAT Meeting Rep Ser 1 Rome Italy. 10.4060/cc0178en

[CR27] FAO (2024) Programme Against African Trypanosomosis (PAAT), The Disease. Food and Agriculture Organization of the United Nations (FAO). Available: http://www.fao.org/paat/the-pro-gramme/the-disease/en/. Accessed 15 Sept 2024

[CR28] Flamini G, Tebano M, Cioni PL, Bagci Y, Dural H, Ertugrul K, Uysal T, Savran A (2006) A multivariate statistical approach to *Centaurea* classification using essential oil composition data of some species from Turkey. Pl Syst Evol 261(1–4):217–228. 10.1007/s00606-006-0448-3

[CR29] Franco JR, Priotto G, Paone M, Cecchi G, Ebeja AK, Simarro PP, Sankara D, Metwally SBA, Argaw DD (2024) The elimination of human African trypanosomiasis: Monitoring progress towards the 2021–2030 WHO road map targets. PLoS Negl Trop Dis 18(4):e0012111. 10.1371/journal.pntd.0012111 38626188 10.1371/journal.pntd.0012111 PMC11073784

[CR30] Gaithuma A, Yamagishi J, Hayashida K, Kawai N, Namangala B, Sugimoto C (2020) Blood meal sources and bacterial microbiome diversity in wild-caught tsetse flies. Sci Rep 10:5005. 10.1038/s41598-020-61817-2 32193415 10.1038/s41598-020-61817-2 PMC7081217

[CR31] Gashururu RS, Maingi N, Githigia SM, Getange DO, Ntivuguruzwa JB, Habimana R, Cecchi G, Gashumba J, Bargul JL, Masiga DK (2023) Trypanosomes infection, endosymbionts, and host pref-erences in tsetse flies (Glossina spp) collected from Akagera park region, Rwanda: a correlational xenomonitoring study. One Health 16:100550. 10.1016/j.onehlt.2023.100550 37363264 10.1016/j.onehlt.2023.100550 PMC10288097

[CR32] Gibson G, Torr SJ (1999) Visual and olfactory responses of haema-tophagous Diptera to host stimuli. Med Vet Entomol 13(1):2–23. 10.1046/j.1365-2915.1999.00163.x 10194745 10.1046/j.1365-2915.1999.00163.x

[CR33] Gikonyo NK, Hassanali A, Njagi PG, Saini RK (2000) Behaviour of *Glossina morsitans morsitans* Westwood (Diptera: Glos-sinidae) on waterbuck *Kobus Defassa* Ruppel and feeding membranes smeared with waterbuck sebum indicates the presence of allomones. Acta Trop 77:295–303. 10.1016/s0001-706x(00)00153-4 11114392 10.1016/s0001-706x(00)00153-4

[CR34] Gikonyo NK, Hassanali A, Njagi PGN, Gitu PM, Midiwo JO (2002) Odour composition of preferred (buffalo and ox) and nonpre-ferred (waterbuck) hosts of some Savannah tsetse flies. J Chem Ecol 28:961–973. 10.1023/a:1015205716921 10.1023/a:1015205716921 12049234

[CR35] Gikonyo NK, Hassanali A, Njagi PGN, Saini RK (2003) Responses of *Glossina morsitans morsitans* to blends of electroantennographi-cally active compounds in the odours of its preferred (Buffalo and ox) and none preferred (waterbuck) hosts. J Chem Ecol 29:2331–2346. 10.1023/a:1026230615877 14682515 10.1023/a:1026230615877

[CR36] Guerenstein PG, Lazzari CR (2009) Host-seeking: how triatomines acquire and make use of information to find blood. Acta Trop 110(2–3):148–158. 10.1016/j.actatropica.2008.09.019 18983971 10.1016/j.actatropica.2008.09.019

[CR37] Hervé MR, Nicolè F, Lê Cao K-A (2018) Multivariate analysis of multiple datasets: a practical guide for chemical ecology. J Chem Ecol 44(3):215–234. 10.1007/s10886-018-0932-6 29479643 10.1007/s10886-018-0932-6

[CR38] Insausti K, Goñi V, Petri E, Gorraiz C, Beriain MJ (2005) Effect of weight at slaughter on the volatile compounds of cooked beef from Spanish cattle breeds. Meat Sci 70(1):83–90. 10.1016/j.meatsci.2004.12.003 22063283 10.1016/j.meatsci.2004.12.003

[CR39] Kotowska U, Zalikowski M, Isidorov VA (2012) HS-SPME/GC-MS analysis of volatile and semi-volatile organic compounds emitted from municipal sewage sludge. Environ Monit Asses 184(5):2893–2907. 10.1007/s10661-011-2158-8 10.1007/s10661-011-2158-8 21688031

[CR40] KWS (2024) Kenya Wildlife Service 2024, Amboseli National Park. https://www.kws.go.ke/amboseli-national-park. Accessed 15 Sept 2024

[CR41] Lalel HJD, Singh Z, Chye Tan S (2003) Glycosidically-bound aroma volatile compounds in the skin and pulp of “Kensington Pride” mango fruit at different stages of maturity. Postharvest Biol Technol 29(2):205–218. 10.1016/S0925-5214(02)00250-8

[CR42] Le Cao K-A, Boitard S, Besse P (2011) Sparse PLS discriminant analysis: biologically relevant feature selection and graphical displays for multiclass problems. BMC Bioinform 12:253. 10.1186/1471-2105-12-253 10.1186/1471-2105-12-253 PMC3133555 21693065

[CR43] Liaw A, Wiener M (2002) Classification and regression by Random Forest. R News 2(3):18–22

[CR44] Makhulu EE, Villinger J, Adunga VO, Jeneby MM, Kimathi EM, Mararo E, Oundo JW, Musa AA, Wambua L (2021) Tsetse blood-meal sources, endosymbionts and trypanosome-associations in the Maasai Mara National Reserve, a wildlife-human-livestock interface. PLoS Negl Trop Dis 15(1):e0008267. 10.1371/journal.pntd.0008267 33406097 10.1371/journal.pntd.0008267 PMC7822626

[CR45] Masiga DK, Igweta L, Saini R, Ochieng JP, Borgemeister C (2014) Building endogenous capacity for the management of neglected tropical diseases in Africa: the pioneering role of icipe. PLoS Negl Trop Dis 8(5):e2687. 10.1371/journal.pntd.0002687 24830708 10.1371/journal.pntd.0002687 PMC4022455

[CR46] Miano RN, Ayelo PM, Musau R, Hassanali A, Mohamed SA (2022) Electroantennogram and machine learning reveal a volatile blend mediating avoidance behavior by *Tuta absoluta* females to a wild tomato plant. Sci Rep 12:8965. 10.1038/s41598-022-13125-0 35624177 10.1038/s41598-022-13125-0 PMC9142488

[CR47] Mimica-Dukic N, Kujundzic S, Sokovic M, Couladis M (2003) Essential oil composition and antifungal activity of Foeniculum vulgare Mill. obtained by different distillation conditions. Phytother Res 17(4):368–371. 10.1002/ptr.1159 12722142 10.1002/ptr.1159

[CR48] Moloo SK (1993) The distribution of *Glossina* species in Africa and their natural hosts. Int J Trop Insect Sci 14(4):511–527. 10.1017/S1742758400014211

[CR49] Muriithi BW, Gathogo NG, Diiro GM, Kidoido MM, Okal MN, Masiga DK (2021) Farmer perceptions and willingness to pay for novel livestock pest control technologies: a case of tsetse repellent collar in Kwale County in Kenya. PLoS Negl Trop Dis 15(8):e0009663. 10.1371/journal.pntd.0009663 34403426 10.1371/journal.pntd.0009663 PMC8396722

[CR50] Muturi CN, Ouma JO, Malele II, Ngure RM, Rutto JJ, Mithöfer KM, Enyaru J, Masiga DK (2011) Tracking the feeding patterns of tse-tse flies (*Glossina* Genus) by analysis of bloodmeals using mito-chondrial cytochromes genes. PLoS ONE 6(2):e17284. 10.1371/journal.pone.0017284 21386971 10.1371/journal.pone.0017284 PMC3046180

[CR51] Olaide OY, Tchouassi DP, Yusuf AA, Pirk CW, Masiga DK, Saini RK, Torto B (2019) Zebra skin odor repels the Savannah tsetse fly, *Glossina pallidipes* (Diptera: Glossinidae). PLoS Negl Trop Dis 13:e0007460. 10.1371/journal.pntd.0007460 31181060 10.1371/journal.pntd.0007460 PMC6586361

[CR52] Olaide OY, Tchouassi DP, Yusuf AA, Pirk CW, Masiga DK, Saini RK, Torto B (2021) Effect of zebra skin-derived compounds on field catches of the human African trypanosomiasis vector *Glossina fuscipes fuscipes*. Acta Trop 213:105745. 10.1016/j.actatropica.2020.105745 33160957 10.1016/j.actatropica.2020.105745

[CR53] Oloo F, Sciarretta A, Mohamed-Ahmed MM, Krober T, McMullin A, Mihok S, Guerin PM (2014) Standardizing visual control devices for tsetse flies: East African species *Glossina fuscipes fuscipes* and *Glossina tachinoides*. PLoS Negl Trop Dis 8:e3334. 10.1371/journal.pntd.0003334 25411931 10.1371/journal.pntd.0003334 PMC4239017

[CR54] Orubuloye OY, Mbewe NJ, Tchouassi DP, Yusuf AA, Pirk CW, Torto B (2024) An Overview of Tsetse Fly Repellents: Identification and Applications. J Chem Ecol10.1007/s10886-024-01527-5 10.1007/s10886-024-01527-5 PMC11543710 38976099

[CR55] Ouma JO, Marquez JG, Krafsur ES (2006) Microgeographic breeding structure of the tsetse fly, *Glossina pallidipes* in southwestern Kenya. Med Vet Entomol 20(1):138–149. 10.1111/j.1365-2915.2006.00609.x 16608498 10.1111/j.1365-2915.2006.00609.x PMC1450340

[CR56] Paul E, Sikes RS, Beaupre SJ, Wingfield JC (2016) Animal Welfare Policy: Implementation in the Context of Wildlife Research—Policy Review and Discussion of Fundamental Issues. ILAR J 56(3):312–334. 10.1093/ilar/ilv073 26912718 10.1093/ilar/ilv073

[CR57] Peng CT, Hua RL, Maltby D (1992) Prediction of retention indexes: IV. Chain branching in alkylbenzene isomers with C10–13 alkyl chains identified in a scintillator solvent. J Chromatogr 589(1–2):231–239. 10.1016/0021-9673(92)80027-R 1541662 10.1016/0021-9673(92)80027-r

[CR58] Pino JA, Mesa J, Muñoz Y, Martí MP, Marbot R (2005) Volatile components from mango (Mangifera indica L.) cultivars. J Agric Food Chem 53(6):2213–2223. 10.1021/jf0402633 15769159 10.1021/jf0402633

[CR59] R Core Team (2024) R: a language and environment for statistical computing. R Foundation for Statistical Computing, Vienna. https://www.R-project.org/. Accessed 15 Sept 2024

[CR60] Radulovic NS, Blagojevic PD, Palic RM, Zlatkovic BK, Stevanovic BM (2009) Volatiles from vegetative organs of the paleoendemic resurrection plants *Ramonda serbica* Panc. and *Ramonda nathaliae* Panc. at Petrov. J Serb Chem Soc 74(1):35–44. 10.2298/JSC0901035R

[CR61] Ranganathan Y, Borges RM (2010) Reducing the babel in plant volatile communication: using the forest to see the trees. Plant Biol 12(5):735–742. 10.1111/j.1438-8677.2009.00278.x 20701696 10.1111/j.1438-8677.2009.00278.x

[CR62] Rayaisse JB, Tirados I, Kaba D, Dewhirst SY, Logan JG, Diarrassouba A, Salou E, Omolo MO, Solano P, Lehane MJ, Pickett JA, Vale GA, Torr SJ, Esterhuizen J (2010) Prospects for the develop-ment of odour baits to control the tsetse flies *Glossina tachinoi-des* and *G* Palpalis s.l. PLoS Negl Trop Dis 4:e632. 10.1371/journal.pntd.0000632 20300513 10.1371/journal.pntd.0000632 PMC2838779

[CR63] Rohart F, Gautier B, Singh A, Le Cao K-A (2017) MixOmics: An R package for ‘Omics feature selection and multiple data integration. PLOS Comput Biol 13:e1005752. 10.1371/journal.pcbi.1005752 29099853 10.1371/journal.pcbi.1005752 PMC5687754

[CR64] Saini RK, Orindi BO, Mbahin N, Andoke JA, Muasa PN, Mbuvi DM, Muya CM, Pickett JA, Borgemeister CW (2017) Protecting cows in small-holder farms in East Africa from tsetse flies by mimick-ing the odour profile of a non-host bovid. PLoS Negl Trop Dis 11:e0005977. 10.1371/journal.pntd.0005977 29040267 10.1371/journal.pntd.0005977 PMC5659797

[CR65] Saroglou V, Marin PD, Rancic A, Veljic M, Skaltsa H (2007) Composition and antimicrobial activity of the essential oil of six *Hypericum* species from Serbia. Biochem Syst Ecol 35(3):146–152. 10.1016/j.bse.2006.09.009

[CR66] Sartin JH, Halsall CJ, Davison B, Owen S, Hewitt CN (2001) Determination of biogenic volatile organic compounds (C_8_–C_16_) in the coastal atmosphere at Mace Head. Ireland Anal Chim Acta 428(1):61–72. 10.1016/S0003-2670(00)01214-9

[CR67] Shaw APM, Cecchi G, Wint GRW, Cecchi G, Torr S, Waiswa C, Temesgen A, Eregae M, Abdi A, Muchina S, Mugasi S, Mattioli R, Mattioli RC, Robinson TP (2017) Intervening against bovine trypanosomosis in Eastern Africa: mapping the costs and benefits: mapping the costs and benefits. Programme Against African Try-panosomosis, Food and Agriculture Organistaion of the United Nations, Rome, Italy. Available: http://www.fao.org/3/a-i7342e. pdf. Accessed 15 Sept 2024

[CR68] Skaltsa HD, Mavrommati A, Constantinidis T (2001) A chemotaxonomic investigation of volatile constituents in Stachys subsect. Swainsonianeae (Labiatae). Phytochemistry 57(2):235–244. 10.1016/S0031-9422(01)00003-6 11382239 10.1016/s0031-9422(01)00003-6

[CR69] Slijkerman RW, Song F, Astuti GD, Huynen MA, van Wijk E, Stieger K, Collin RW (2015) The pros and cons of vertebrate animal models for functional and therapeutic research on inherited retinal dystrophies. Prog Retin Eye Res 48:137–159. 10.1016/j.preteyeres.2015.04.004 25936606 10.1016/j.preteyeres.2015.04.004

[CR70] Stanczyk NM, De Moraes CM, Mescher MC (2018) Can we use human odors to diagnose malaria? Future Microbiol 14:5–9. 10.2217/fmb-2018-0312 30565951 10.2217/fmb-2018-0312 PMC6939219

[CR71] Takken W (1991) The Role of Olfaction in Host-Seeking of Mosquitoes: A Review. Int J Trop Insect Sci 12:287–295. 10.1017/S1742758400020816

[CR72] Takken W, Knols BG (2010) Strategic use of chemical ecology for vector-borne disease control. In: Takken W, Knols BG (eds) Olfaction in vector-host interactions, vol 2. Wageningen Academic Publishers, Wageningen, pp 13–16

[CR73] Tchouassi DP, Sang R, Sole CL, Bastos ADS, Teal PEA, Borgemeister C, Torto B (2013) Common host-derived chemicals increase catches of disease-transmitting mosquitoes and can improve early warning systems for Rift Valley Fever Virus. PLoS Negl Trop Dis 7(1):e2007. 10.1371/journal.pntd.0002007 23326620 10.1371/journal.pntd.0002007 PMC3542179

[CR74] Tchouassi DP, Milugo TK, Torto B (2024) Feasibility of sand fly control based on knowledge of sensory ecology. Curr Opin Insect Sci 66:101274. 10.1016/j.cois.2024.101274 39341456 10.1016/j.cois.2024.101274

[CR75] Thioulouse J, Dray S, Dufour A, Siberchicot A, Jombart T, Pavoine S (2018) Multivariate Analysis of Ecological Data with ade4. Springer. 10.1007/978-1-4939-8850-1

[CR76] Torr SJ, Vale GA (2015) Know your foe: lessons from the analysis of tsetse fly behavior. Trends Parasitol 31:95–99. 10.1016/j.pt.2014.12.010 25599585 10.1016/j.pt.2014.12.010

[CR77] Torr SJ, Mangwiro TNC, Hall DR (2006) The effects of host physiology on the attraction of tsetse (Diptera: Glossinidae) and Stomoxys (Diptera: Muscidae) to cattle. Bull Entomol Res 96(01):71–84. 10.1079/BER2005404 16441907 10.1079/ber2005404

[CR78] Torr SJ, Prior A, Wilson PJ, Schofield S (2007) Is there safety in numbers? The effect of cattle herding on biting risk from tsetse flies. Med Vet Entomol 21(4):301–311. 10.1111/j.1365-2915.2007.00705.x 18092968 10.1111/j.1365-2915.2007.00705.x

[CR79] Turchini GM, Giani I, Caprino F, Moretti VM, Valfrè F (2004) Discrimination of origin of farmed trout by means of biometrical parameters, fillet composition and flavor volatile compounds. Ital J Anim Sci 3(2):123–140. 10.4081/ijas.2004.123

[CR80] UNESCO (2024) United Nations Educational, Scientific and Cultural Organization (UNESCO) World Heritage Convention, UNESCO World Heritage Centre 1992–2024. Available: https://whc.unesco.org/en/tentativelists/6656/. Accessed 15 Sept 2024

[CR81] Vagionas K, Ngassapa O, Runyoro D, Graikou K, Gortzi O, Chinou I (2007) Chemical analysis of edible aromatic plants growing in Tanzania Food Chem 105(4)>1711–1717. 10.1016/j.foodchem.2007.05.029

[CR82] Vale GA, Hall DR, Gough AJE (1988) The olfactory responses of tsetse flies, Glossina spp. (Diptera: Glossinidae), to phenols and urine in the field. Bull Entomol Res 78(2):293–300. 10.1017/S0007485300013055

[CR83] Vale GA, Hargrove JW, Solano P, Courtin F, Rayaisse JB, Lehane MJ, Esterhuizen J, Tirados I, Torr SJ (2014) Explaining the host-finding behavior of blood-sucking insects: computerised simulation of the effects of habitat geometry on tsetse fly movement. PLoS Negl Trop Dis 8:e2901. 10.1371/journal.pntd.0002901 24921243 10.1371/journal.pntd.0002901 PMC4055578

[CR84] Vale GA, Torr SJ (2004) Development of bait technology to control tsetse. In: Maudlin I, Holmes PH, Miles MA (eds) The Trypano-somiases. CABI Publishing Wallingford, Oxfordshire, UK, pp 509–524.10.1079/9780851994758.0509

[CR85] van den Dool H, Kratz P (1963) A generalization of the retention index system including linear temperature programmed gas–liquid partition chromatography. J Chromatogr A 11:463–471. 10.1016/S0021-9673(01)80947-X 10.1016/s0021-9673(01)80947-x 14062605

[CR86] Verderio P, Lecchi M, Ciniselli CM, Shishmani B, Apolone G, Manenti G (2023) 3Rs Principle and Legislative Decrees to Achieve High Standard of Animal Research Animals 13(2):277. 10.3390/ani13020277 10.3390/ani13020277 PMC9854901 36670818

[CR87] Vreysen MJB, Seck MT, Sall B, Bouyer J (2013) Tsetse flies: Their biology and control using area-wide integrated pest manage-ment approaches. J Invertebr Pathol 112:S15-25. 10.1016/j.jip.2012.07.026 22878217 10.1016/j.jip.2012.07.026

[CR88] Walzer C (2007) Non domestic equids. In: West G, Heard D, Caulkett N (eds) Zoo animal and wildlife immobilization and anesthesia. Blackwell Publishing Professional, Ames, pp 523–531

[CR89] Xu X, van Stee LLP, Williams J, Beens J, Adahchour M, Vreuls RJJ, Brinkman UATh, Lelieveld J (2003) Comprehensive two-dimensional gas chromatography (GC×GC) measurements of volatile organic compounds in the atmosphere. Atmos Chem Phys 3(3):665–682. 10.5194/acp-3-665-2003

[CR90] Yamaguchi K, Shibamoto T (1981) Volatile constituents of green tea, Gyokuro (Camellia sinensis L var Yabukita). J Agric Food Chem 29(2):366–370. 10.1021/jf00104a035

[CR91] Zeng Y-X, Zhao C-X, Liang Y-Z, Yang H, Fang H-Z, Yi L-Z, Zeng Z-D (2007) Comparative analysis of volatile components from *Clematis* species growing in China. Anal Chim Acta 595(1–2):328–339. 10.1016/j.aca.2006.12.022 17606017 10.1016/j.aca.2006.12.022

[CR92] Zenkevich IG (2005) Experimentally measured retention indices. https://webbook.nist.gov/cgi/cbook.cgi?ID=C106514&Units=SI&Mask=2000#ref-2

